# Biomarker identification and pathway analysis of *Astragalus membranaceus* and *Curcuma zedoaria* couplet medicines on adenine-induced chronic kidney disease in rats based on metabolomics

**DOI:** 10.3389/fphar.2023.1103527

**Published:** 2023-04-06

**Authors:** Lingfei Lu, Jiandong Lu, Jiwei Chen, Bing Wang, Hongcheng Peng, Jinting Peng, Xinhui Liu, Feng Lin, Guoliang Xiong

**Affiliations:** ^1^ Department of Nephrology, Shenzhen Traditional Chinese Medicine Hospital, Guangzhou University of Chinese Medicine, Shenzhen, Guangdong, China; ^2^ The Fourth Clinical Medical College of Guangzhou University of Chinese Medicine, Shenzhen, Guangdong, China; ^3^ Department of Nephrology, Shenzhen Traditional Chinese Medicine Hospital Nanjing University of Chinese Medicine, Shenzhen, China; ^4^ Department of Gynecology, Shenzhen Traditional Chinese Medicine Hospital, Guangzhou University of Chinese Medicine, Shenzhen, Guangdong, China; ^5^ Department of Urology, Shenzhen Traditional Chinese Medicine Hospital Guangzhou University of Chinese Medicine, Shenzhen, Guangdong, China

**Keywords:** *Astragalus membranaceus*, *Curcuma zedoaria*, chronic kidney disease, metabolomic, SIRT1/JNK signaling pathway

## Abstract

**Background:** Chronic kidney disease (CKD) is usually insidious, and most affected individuals are asymptomatic until the disease becomes advanced. The effective treatment of CKD would rely on the incorporation of multidisciplinary approaches. *Astragalus membranaceus* (AM) and *Curcuma zedoaria* (CZ) have been widely used in the treatment of CKD. However, the mechanism of AM and CZ in the treatment of CKD is still unclear.

**Methods:** This study was designed to evaluate the effects of AM and CZ on adenine-induced rats and to investigate the underlying mechanism by using metabolomic analysis. Addition of 0.75% adenine to the diet of rats for 3 weeks induced the animal model of CKD. The rats in the treatment group were treated with AM and CZ (2.1 g/kg/day) for 4 weeks. Blood and kidney samples were collected for biochemical and histological examination. Ultra-high-performance liquid chromatography/Q Exactive HFX mass spectrometer (UHPLC-QE-MS) was applied to analyze metabolic profiling variations in the kidney.

**Results:** The results showed that AM and CZ could significantly reduce serum creatinine (Scr) and blood urea nitrogen (BUN) levels in CKD rats and alleviate renal pathological injury. By comparing the endogenous components of the normal group and the model group in positive ion mode and negative ion mode, a total of 365 and 155 different metabolites were screened, respectively. A total of 117 and 73 metabolites with significantly different expressions were identified between model group and AM and CZ group in positive ion mode and negative ion mode, respectively. The pivotal pathways affected by AM and CZ included nicotinate and nicotinamide metabolism, and glycine, serine and threonine metabolism. Furthermore, significant changes in metabolites in CKD rats after AM and CZ therapies were observed, including L-Threonine, D-pantothenic acid, and nicotinamide. Moreover, we found that AM and CZ significantly reduced renal fibrosis and inflammation in CKD rats, which may be related to the regulation of SIRT1/JNK signaling pathway.

**Conclusion:** In conclusion, AM and CZ significantly reduced renal fibrosis and inflammation in CKD rats, which may be related to the regulation of SIRT1/JNK signaling pathway. Furthermore, L-Threonine, D-pantothenic acid, and nicotinamide may be potential biomarkers for the progression and treatment of CKD.

## Introduction

Chronic kidney disease (CKD) is a progressive disease with a high mortality and morbidity rate. In 2017, there were 697.5 million cases of CKD globally, and the CKD mortality rate for all ages increased by 41·5% from 1990 to 2017 (Bikbov et al., 2020). According to WHO Global Health Estimates, kidney diseases are the world’s 10th leading cause of death. Mortality has increased from 813,000 in 2,000 to 1.3 million in 2019. The burden of CKD is increasing worldwide. CKD is usually caused by diabetes and hypertension (Kalantar-Zadeh et al., 2021). Optimal management of CKD involves reducing cardiovascular risk, treating albuminuria, avoiding nephrotoxins, and adjusting drug dosage (Chen et al., 2019). Even though treatment methods such as the angiotensin converting enzyme inhibitor (ACEI) or angiotensin receptor blocker (ARB) are available (Inker et al., 2014) and, more recently, a sodium-glucose cotransporter 2 (SGLT2) (Cherney et al., 2020; Heerspink et al., 2020) for the treatment of diabetic and non-diabetic CKD patients, risk of CKD progression remains high (Perkovic et al., 2019), and new therapies are needed.

Traditional Chinese Medicine (TCM) has been recognized as a potentially effective therapy for CKD (Zhong et al., 2013; Deng et al., 2020; Xu et al., 2021). *Astragalus membranaceus* (AM) has been clinically applied to treat CKD for several years due to its immunosuppressive, anti-fibrotic, and anti-inflammatory properties (Xu et al., 2016; Chen et al., 2020; Hu et al., 2020; Han et al., 2021). *Curcuma zedoaria* (CZ) has a potential to prevent and/or manage various diseases due to its anti-inflammatory, antioxidant, and anti-apoptotic properties (White et al., 2019; Alvarenga et al., 2020; Patel et al., 2020). Couplet medicines, one of the unique characteristics of TCM, are a combination of two single herbs used in tandem to treat a specific disease to increase effectiveness or minimize side effects (Chan, 1995). The combination of AM and CZ has been widely used in the treatment of CKD in TCM, but their combination mechanism is not clear.

Metabonomics has emerged as an important technology to understand the pathophysiology, molecular mechanism and identify diagnosis and prognosis of various diseases (Kalantari and Nafar, 2019). Metabolomics has been increasingly used in exploring biomarkers and mechanisms of CKD (Wang et al., 2019; Dubin and Rhee, 2020; Hunter et al., 2021). In this study, we undertook a metabolomics analysis to explore the metabolic profiles and identify potential biomarkers in CKD rats with AM and CZ treatment.

## Materials and methods

### Materials and reagents

AM and CZ were bought from Guangdong E-Fong Pharmaceutical (Guangdong, China). AM and CZ were standardized to 10 g per bag. They were prepared into a liquid of 1.87 g/mL for intragastric administration. Adenine was obtained from Yuan-ye (Shanghai, China). L-Threonine, D-pantothenic acid, nicotinamide were obtained from Zzstandard (Shanghai, China). Acetonitrile and Methanol were bought from CNW Technologies GmbH (Düsseldorf, Germany). Creatinine Serum Detection Kit and Blood Urea Nitrogen Detection Kit were bought from StressMarq Biosciences (British Columbia, Canada).

### Animals

Male 8-weeks-old Spraque-Dawley (SD) rats (n = 18) were purchased from Guangdong Medical Laboratory Animal Center (Foshan, China, permitted by SCXK,2013-0002 [YUE]). All rats were placed in a facility with fixed temperature (22°C–24°C) and relative humidity (50%–70%) with 12 h light/dark cycle. After 7 days of adaptive feeding, all rats were randomly divided into three groups: normal group (N, n = 6), model group (M, n = 6), and AM and CZ group (AMCZ, n = 6). Addition of 0.75% adenine to the diet of rats for 3 weeks induced the animal model of CKD (Yokozawa et al., 1986; Diwan et al., 2018). The AMCZ was administered AM and CZ (2.1 g/kg/d) by gastric irrigation for 4 weeks. The rats were sacrificed at the end of the experiment, All rats were anesthetized, and blood samples were taken from the abdominal aorta. Kidney samples were collected for further analysis.

### Biochemical analysis

Creatinine Serum Detection Kit (StressMarq Biosciences, British Columbia, Canada) and Blood Urea Nitrogen Detection Kit (StressMarq Biosciences, British Columbia, Canada) were used to measure serum creatinine (SCR) and blood urea nitrogen (BUN).

### Histological analysis

Kidneys were fixed in 4% paraformaldehyde for 24 h and embedded in paraffin. Periodic-acid-Schiff (PAS) staining was performed to evaluate renal pathological injury. Masson staining was used to estimate renal fibrosis. The tubular injury score was calculated using PAS staining and was based on the atrophy, shedding, and tubular dilation of tubular epithelial cells. The scoring system was as follows: 0 = no tubular injury; 1 = <10%; 2 = 10–25%; 3 = 26–50%; 4 = 51–75%; and 5 = >75% tubular injury (Wei et al., 2022). We used ImageJ software (National Institutes of Health, United States) to evaluate tubular interstitial fibrosis in Masson staining. Collagen volume fraction was calculated as follows: collagen area/total area × 100%.

### UHPLC-QE-MS based untargeted metabolomics analysis

The samples were extracted by 500 μL extraction solution (methanol/acetonitrile/water, 2:2:1, with isotopically-labelled internal standard mixture). After the centrifugation (12,000 r/min, 4°C, 15 min), supernatant was transferred into the liquid vial for metabolomics analysis. In order to guarantee the stability and repeatability of the ultra-high-performance liquid chromatography-tandem mass spectrometry (UHPLC-MS/MS) system, we prepared quality control (QC) samples by mixing equal aliquots of the supernatants from each group in the manner described above. The QC samples were staggered with the other samples (after every six samples).

We performed liquid chromatography/tandem mass spectrometry (LC-MS/MS) analysis using a UHPLC system (Vanquish, Thermo Fisher Scientific) coupled to a Q Exactive HFX mass spectrometer (Orbitrap MS, Thermo). The mobile phase consisted of 25 mmol/L ammonium acetate and 25 ammonia hydroxide in water (pH = 9.75) (A) and acetonitrile (B). The acquisition software continuously evaluates the MS spectrum in this mode. ESI source conditions were set as follows: sheath gas flow rate was 30 Arb, aux gas flow rate was 25 Arb, capillary temperature was 350°C, full MS resolution was 60,000, MS/MS resolution was 7,500, collision energy was 10/30/60 in NCE mode, and spray voltage was 3.6 kV (positive) or −3.2 kV (negative).

### Data processing, multivariate data analysis and biomarker identification

The raw data were converted to the mzXML format then using Proteo Wizard and processed with an in-house program, which was developed using R and based on XCMS, for peak detection, extraction, alignment, and integration. After that, an in-house MS2 database (BiotreeDB) was applied to the annotation of metabolites. The cutoff for annotation was set at 0.3. SIMCA software (V15.0.2, Sartorius Stedim Data Analytics AB, Umea, Sweden) was utilized for principal component analysis (PCA) and orthogonal partial least squares discriminant analysis (OPLS-DA). The metabolites with variable importance in projection (VIP) > 1 (OPLS-DA) and *p* < 0.05 (Student’s t-test) were considered as significantly changed metabolites. Additionally, pathway enrichment analyses were conducted using biochemical databases including KEGG (http://www.genome.jp/kegg/) and MetaboAnalyst (http://www.metaboanalyst.ca/).

### UHPLC-MRM-MS/MS analysis

The samples were extracted by 500 μL extraction solution (methanol/acetonitrile/water, 2:2:1, precooling at −40°C). The upon mixture was vortexed for 30s and sonicated for 5 min at 0°C, followed by homogenization for 4 min at 40 Hz. The homogenate and sonicate circle was repeated three times. After the above procedure, the sample was incubated at −40°C for an hour and centrifuged at 12,000 rpm for 15 min at 4°C. The UHPLC separation was carried out using an ExionLC-Sciex (Sciex), equipped with a Waters ACQUITY UPLC BEH Amide (100 × 2.1 mm, 1.7 μm, Waters). The mobile phase A was 5 mM ammonium acetate& 0.1% acetic acid in water, and the mobile phase B was acetonitrile. A SCIEX 6500 QTRAP + triple quadrupole mass spectrometer (Sciex), equipped with an IonDrive Turbo V electrospray ionization (ESI) interface, was applied for assay development. Typical ion source parameters were: Curtain Gas = 40 psi, IonSpray Voltage = ±4500 V, temperature = 475°C, Ion Source Gas 1 = 30 psi, Ion Source Gas 2 = 30 psi. The MRM parameters for each of the targeted analytes were optimized using flow injection analysis and were performed by injecting the standard solutions of the individual analytes into the API source of the mass spectrometer. SCIEX Analyst Work Station Software (Version 1.6.3) and Sciex MultiQuant™ 3.0.3 were employed for MRM data acquisition and processing.

### Western blotting

The cortex tissues of the kidneys were lysed with RIPA lysis buffer. The proteins were separated by electrophoresis, and the proteins on the SDS-polyacrylamide gel were transferred to a polyvinylidene difluoride membrane (Millipore, United States). Following blocking in 5% non-fat milk for 1 h at room temperature, the membranes were incubated with various primary antibodies at 4°C overnight. The primary antibodies included SIRT1(1:1000, proteintech), p-JNK(1:1000, proteintech), JNK(1:1000, proteintech), Fibronectin (FN) (1:1,000, Abcam), Collagen IV(Col-IV) (1:500, Abcam), α-SMA (1:500, Sigma), GAPDH (1:1,000, Cell Signaling Technology),Then, the membranes were incubated in HRP-conjugated secondary antibodies for 1 h at room temperature. HRP activity was visualized using Tanon-6100C.

### Real-time reverse transcription polymerase chain reaction (RT-PCR) assay

Total RNA from the cortex tissues was extracted using RNA Isolation Kit V2 (Vazyme, China), and cDNA was synthesised using the HiScript II Q RT SuperMix for qPCR (+gDNA wiper) (Vazyme, China), according to the manufacturer’s instructions. Real-time RT-PCR was carried out on the cDNA samples using ChamQ Universal SYBR qPCR Master Mix (Vazyme, China). The sequences of primers were as follows: IL-1β forward, 5′-AGC​TCT​CCA​CCT​CAA​TGG​AC-3′ and reverse, 5′-TTG​TTT​GGG​ATC​CAC​ACT​CTC​C-3′, TNF-α forward, 5′-ATG​GGC​TCC​CTC​TCA​TCA​GTT​CC-3′ and reverse, 5′-CCT​CCG​CTT​GGT​GGT​TTG​CTA​C-3′, β-actin forward, 5′-GTG​AAA​AGA​TGA​CCC​AGG​ACT-3′ and reverse, 5′-TCTCATCTGGGAAAGAGCAGAA-3′.All amplifications were carried out in triplicate and repeated three times. The PCR data were analysed using the CFX Connect Real-Time PCR System (Bio-Rad, United States). Cycle threshold (CT) values were analysed using the comparative CT (ΔΔCT) method, and the relative amount of target mRNA (2^−ΔΔCT^) was obtained by normalising to endogenous β-actin levels.

### Statistical analysis

All quantitative were presented as the mean ± SEM. All statistical analyses were performed using GraphPad Prism version 8.0.0 for Windows (GraphPad Software, San Diego, California United States). Statistically significant differences were determined by one-way ANOVA followed by Tukey’s multiple comparisons test. Brown-Forsythe and Welch ANOVA tests when variances are unequal. If the data does not follow a normal distribution, use the Kruskal-Wallis test. Statistical significance was considered at *p* < 0.05.

## Results

### AM and CZ ameliorated renal injury in CKD rat

**FIGURE 1:**
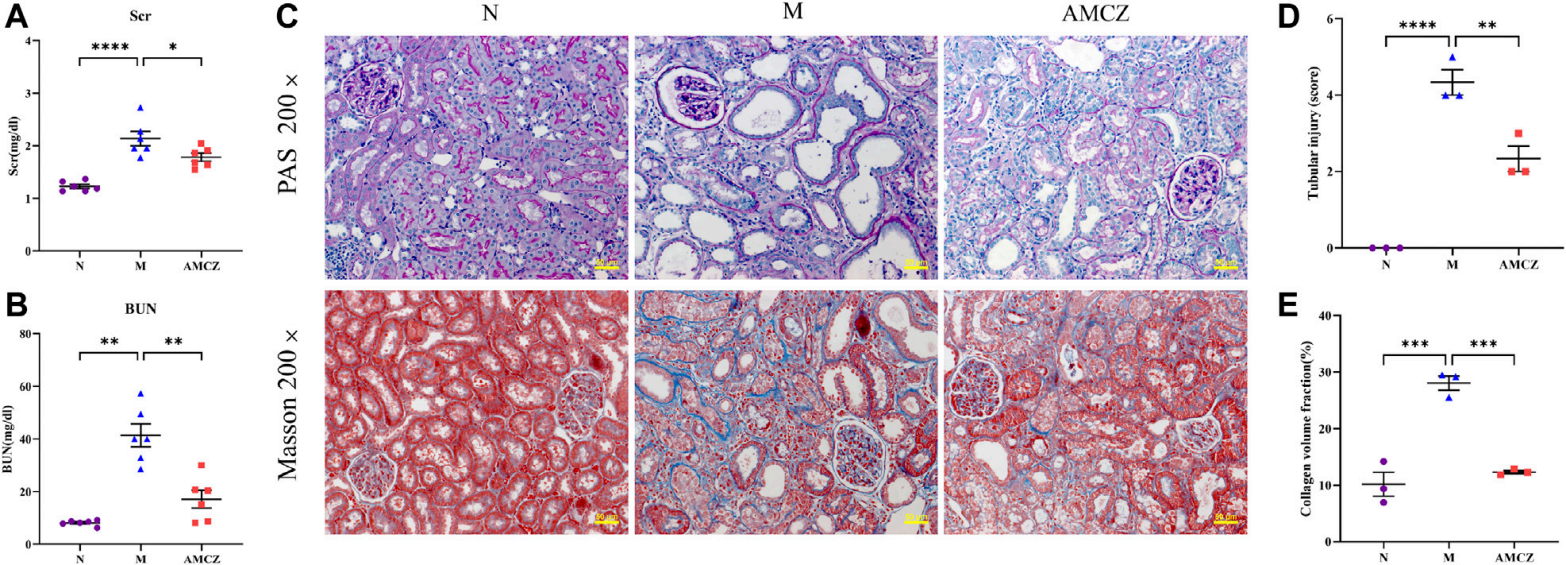
Effects of AM and CZ in the CKD rats. **(A)** The level of Scr (n = 6). **(B)** The level of BUN (n = 6). **(C)** PAS staining and Masson staining of kidney. **(D)** Quantitative analysis of renal tubular injury score in all groups. **(E)** Quantification of collagen volume fraction (collagen area/total area × 100%) in all groups. Data are presented as the means ± SEM (****p < 0.0001, ***p < 0.001, **p < 0.01, *p < 0.05)

The levels of Scr and BUN were significantly increased in model group relative to normal group. This increase was reversed by AM and CZ treatment of the CKD rats (Figures 1A, B). Furthermore, Periodic Acid–Schiff (PAS) staining and Masson staining showed obvious features of CKD, such as tubular atrophy, interstitial fibrosis, and this was significantly inhibited by AM and CZ in CKD rats (Figures 1C–E).

### UHPLC-QE-MS analysis and multivariate analysis of UHPLC-QE-MS data

**FIGURE 2:**
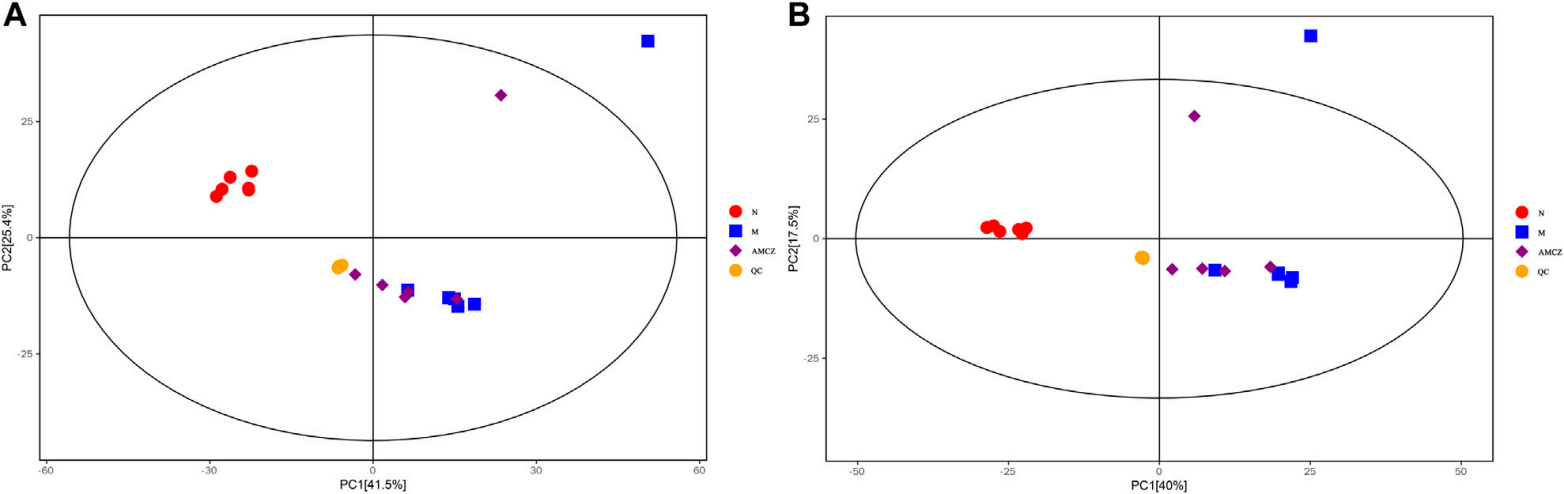
PCA score plots of three groups and QC samples. **(A)** positive ion mode; **(B)** negative ion mode.

**FIGURE 3:**
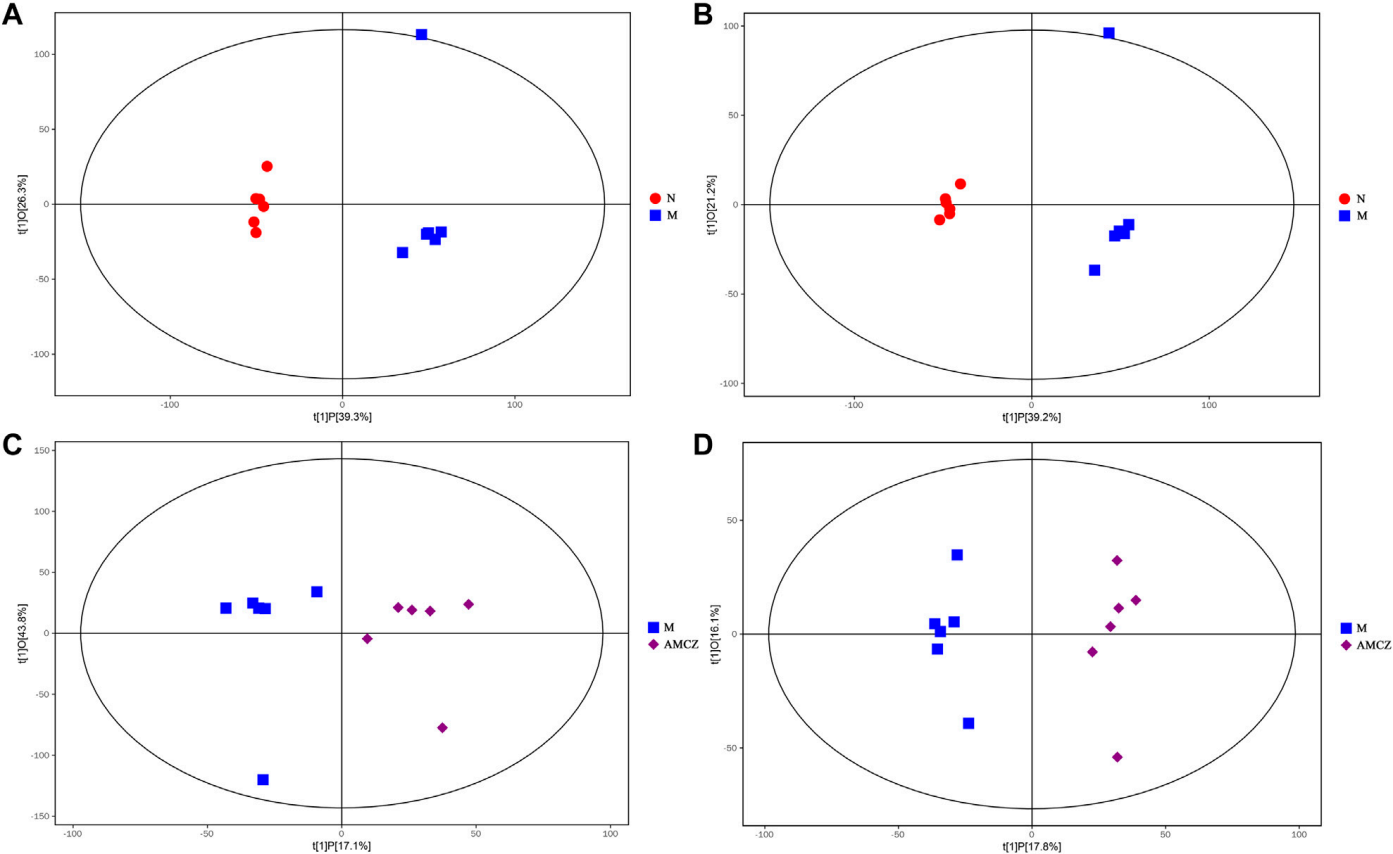
OPLS-DA score plot between three groups. OPLS-DA score plot for N versus M in positive **(A)** and negative **(B)** ion mode. OPLS-DA score plot for M versus AMCZ in positive **(C)** and negative **(D)** ion mode.

UHPLC-QE-MS was used to analyze the kidney samples of normal group (N), model group (M), and AM and CZ group (AMCZ), and data acquisition was performed in positive and negative ion modes. QC samples were used to confirm the reliability of the analytical method, the extracted ion chromatogram (EIC) of QC samples are shown in Supplementary Figure S1. PCA, an unsupervised analysis that reduces the dimensions of data, was applied to visualize the distribution and grouping of the samples. To identify potential outliers in the dataset, the 95% confidence interval in the PCA score plot was used as the threshold. In the PCA of all the samples, we found that QC samples were closely clustered together, which indicated the reliability and stability of the data (Figures 2A, B) and that the obtained data could be used for subsequent metabolomics research. PCA was performed to investigate whether the three groups could be separated and to reveal their metabolic distinctions. As shown in Figures 2A, B, there were distinctly separated clusters between the N, M, and AMCZ groups in both positive ion mode and negative ion mode. A similar finding was also found in the score plots of OPLS-DA, a more reliable pattern recognition method that was carried out to sharpen the separation among groups (Figures 3A–D). In order to examine the robustness and predictive abilities of the OPLS-DA model, 200 permutations were conducted. Then, we obtained the *R* ^2^ and Q^2^ intercept values. *R* ^2^ indicates how well a variable’s variation could be explained, whereas Q^2^ indicates how well a variable could be predicted. The intercept value of Q^2^ represents the robustness of the model, the risk of overfitting, and the degree of reliability of the model. The smaller the intercept of Q^2^, the stronger the robustness (Supplementary Figure S2).

### Identification of potential biomarkers

**FIGURE 4:**
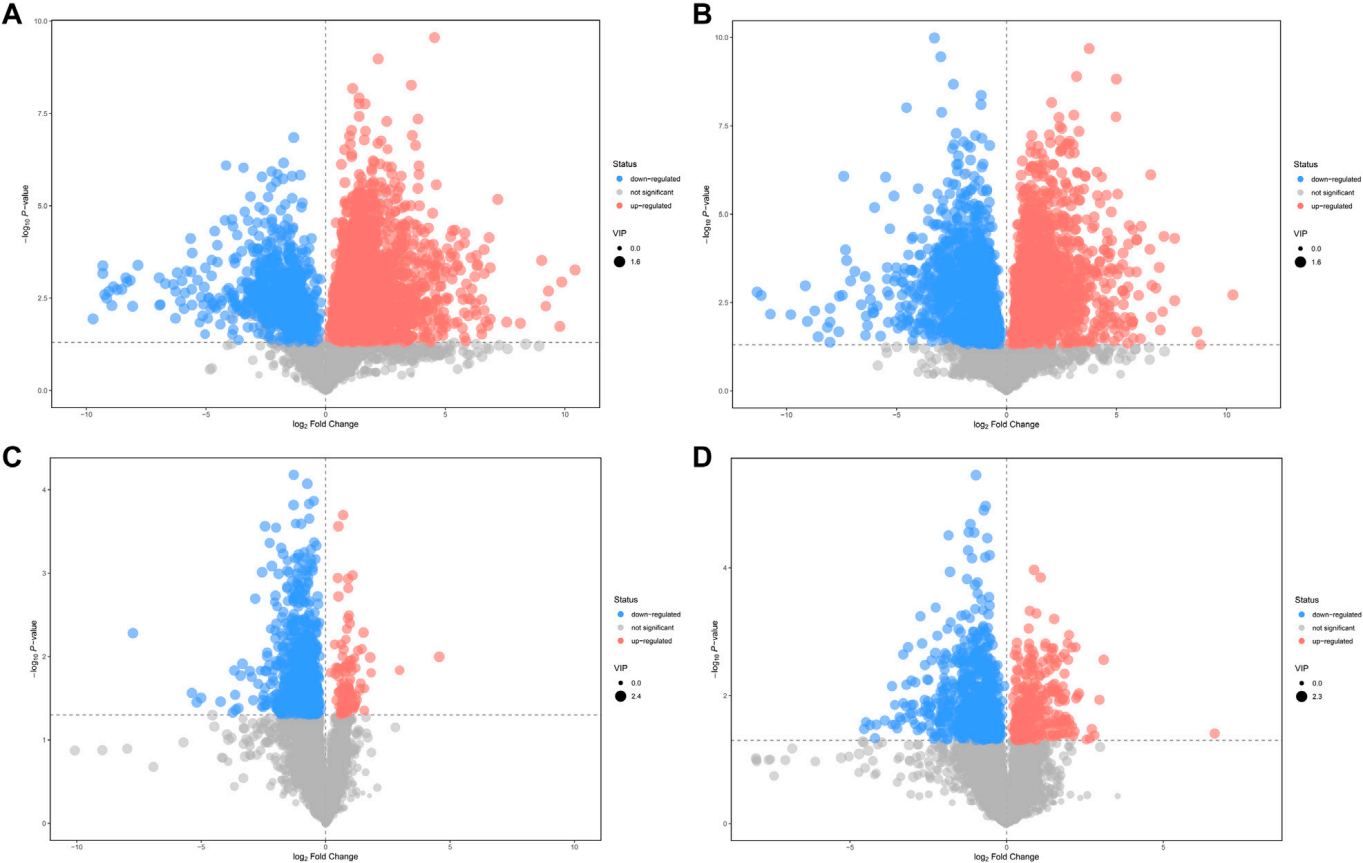
Volcano plots of differential metabolites. Comparison between N and M in positive **(A)** and negative **(B)** ion mode. Comparison between M and AMCZ in positive **(C)** and negative **(D)** ion mode.

A value of variable importance in the projection (VIP) of the first principal component of an OPLS-DA analysis was determined. It indicates the importance of each variable in the model. The metabolites with VIP>1 and *p* < 0.05 were considered as significantly changed metabolites. We visualized and filtered differential metabolites utilizing OPLS-DA mode in the volcano plots (Figure 4). Between N and M group, 365 and 155 metabolites with significant differences were identified under positive ion mode and negative ion mode, respectively (Supplementary Tables S1, S2). In addition, under positive ion mode and negative ion mode, 117 and 73 metabolites with significantly different expressions were identified between M and AMCZ respectively (Supplementary Tables S3, S4).

**FIGURE 5:**
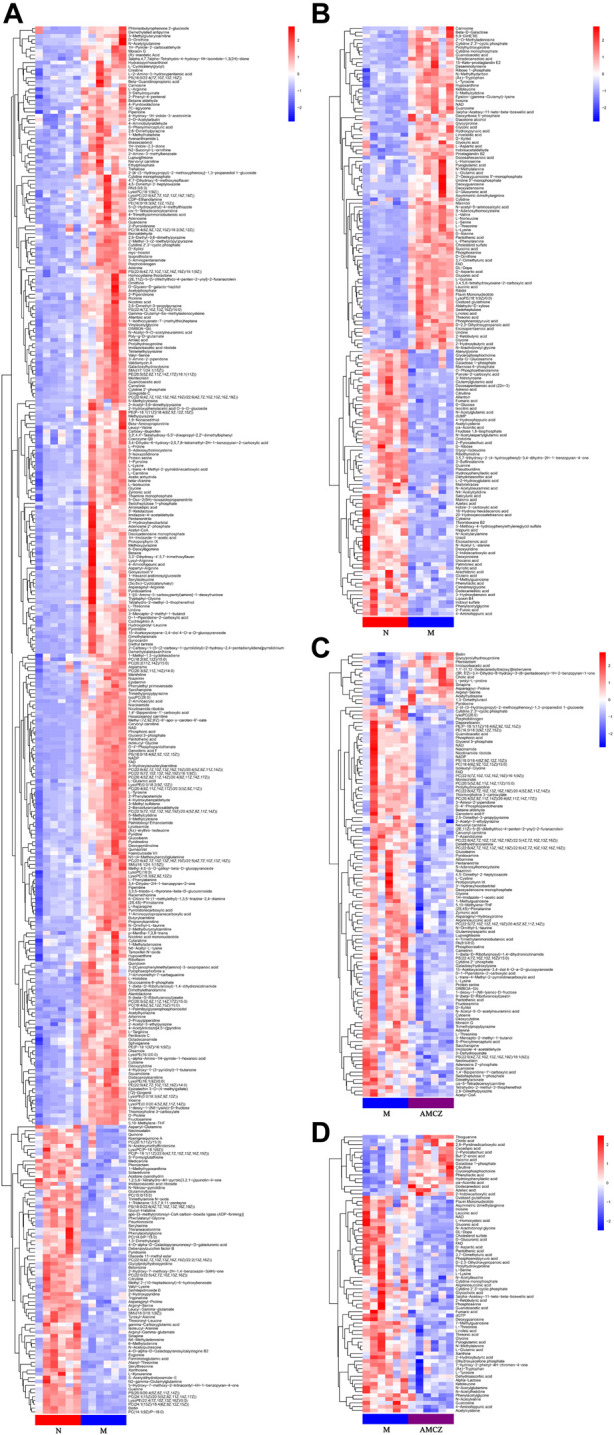
Heatmap of hierarchical clustering analysis of the differential metabolites. Comparison between N and M in positive **(A)** and negative **(B)** ion mode. Comparison between M and AMCZ in positive **(C)** and negative **(D)** ion mode.

**FIGURE 6:**
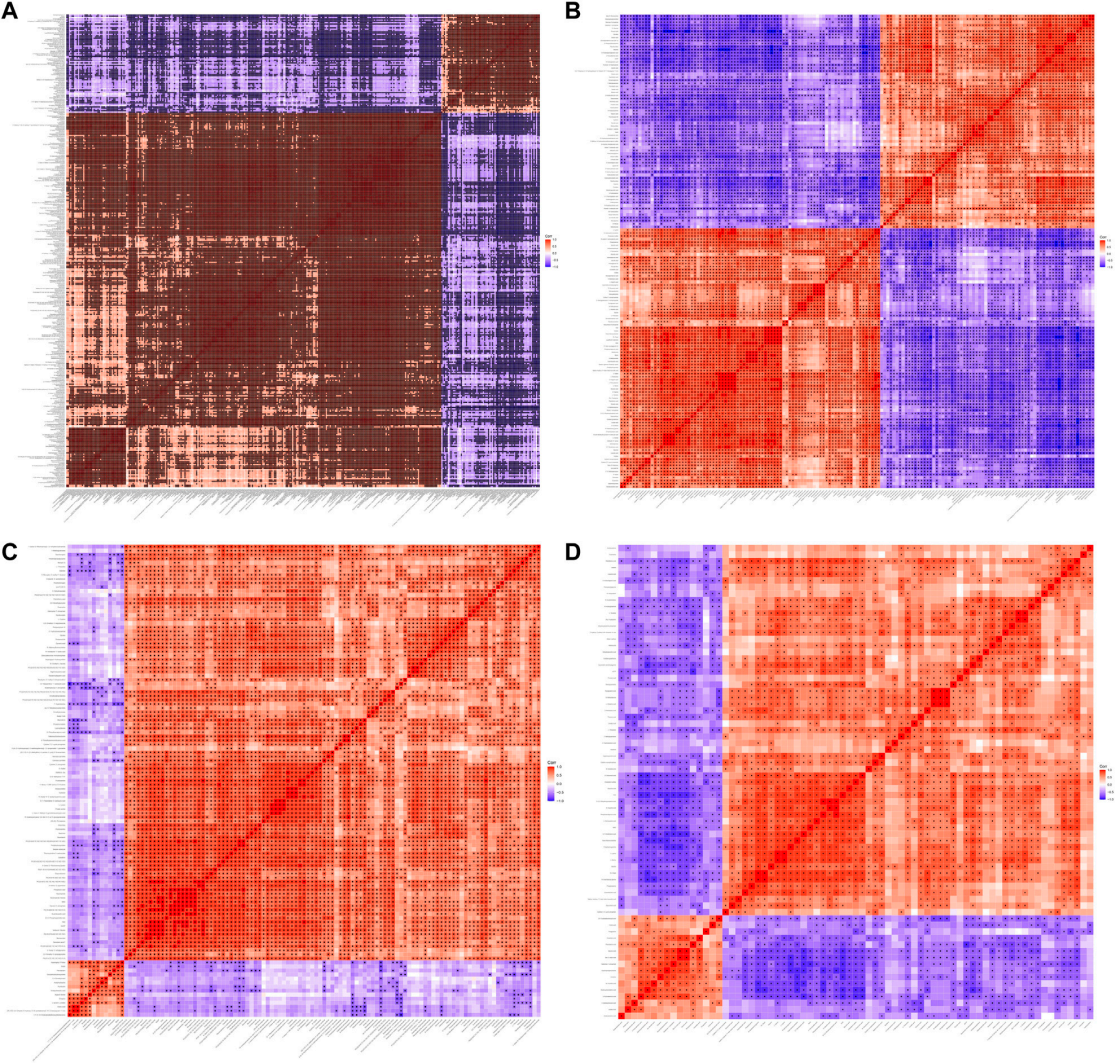
Heatmap of correlation analysis between three groups. Heatmap of correlation analysis N versus M in positive **(A)** and negative **(B)** ion mode. Heatmap of correlation analysis M versus AMCZ in positive **(C)** and negative **(D)** ion mode.

**FIGURE 7:**
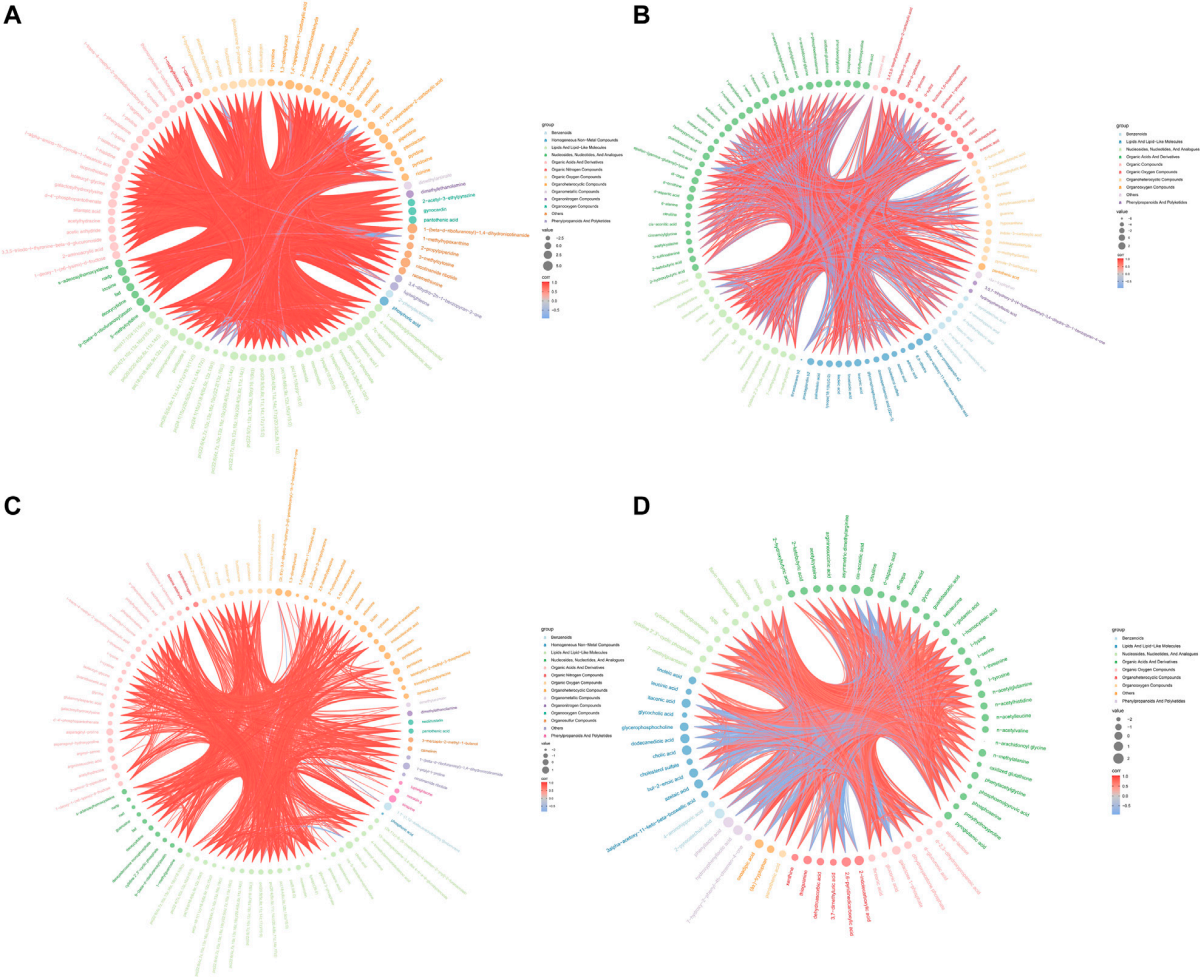
Chord diagram of spearman correlation analysis N versus M in positive **(A)** and negative **(B)** ion mode. Chord diagram of spearman correlation analysis the M versus AMCZ in positive **(C)** and negative **(D)** ion mode.

**FIGURE 8:**
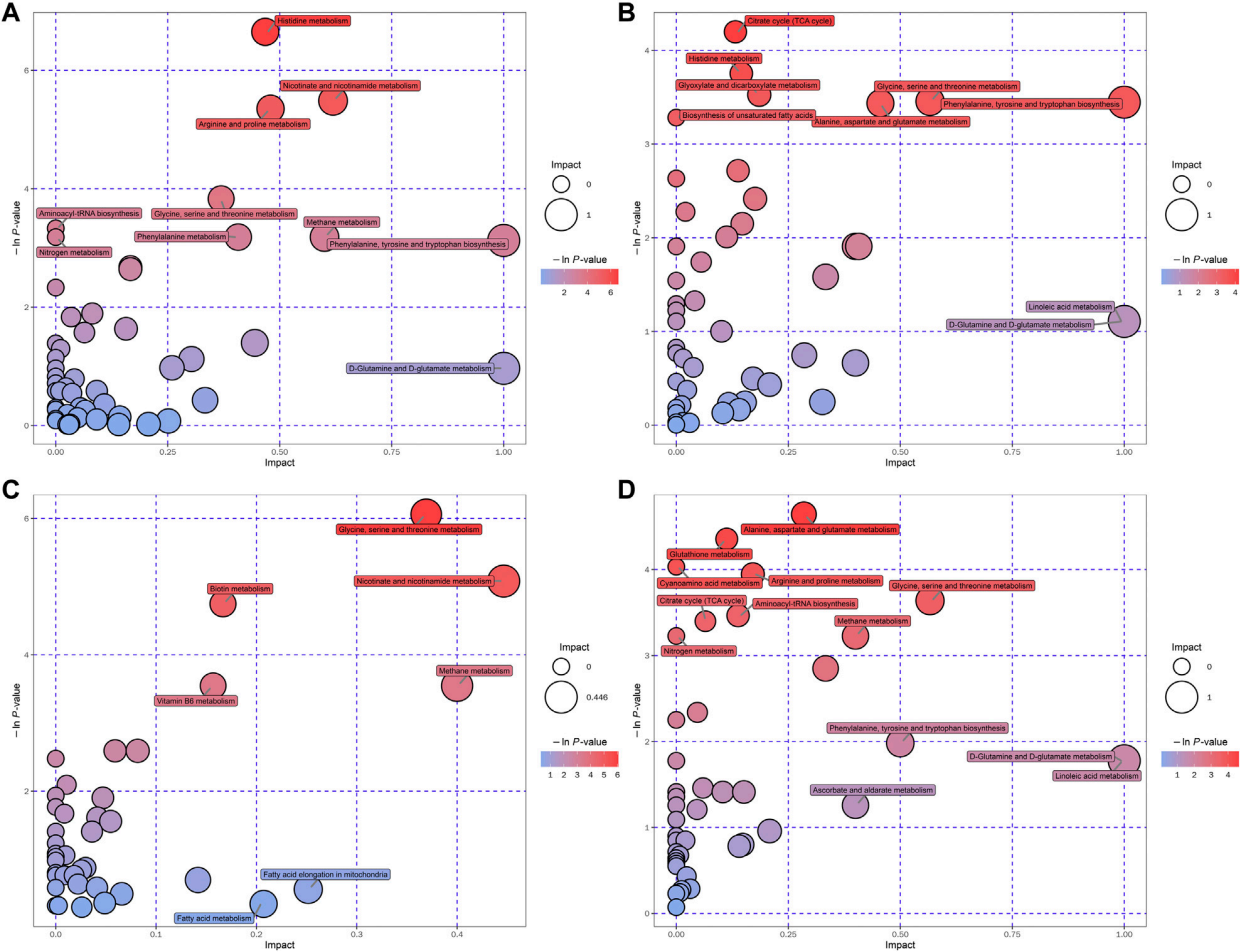
Bubble plots of the metabolic pathway analysis. The main metabolic pathways changed by CKD in negative **(A)** and positive **(B)** ion mode. The main metabolic pathways responded to AM and CZ treatment in negative **(C)** and positive **(D)** ion mode.

Hierarchical clustering analyses and correlation analysis of differential metabolites were shown in Figures 5, 6. Based on the Spearman correlation analysis, the metabolites classification and source were shown by chord diagram. The differential metabolites classification and source between three groups included lipids and lipid like molecules, organoheterocyclic compounds, organic acids and derivatives, nucleosides, nucleotides and analogues, organic acids and derivatives, etc., (Figure 7). Furthermore, we used commercial databases including KEGG (http://www.genome.jp/kegg/) and MetaboAnalyst (http://www.metaboanalyst.ca/) for pathway enrichment analysis. In positive ion mode, the main metabolic pathways significantly affected by CKD included histidine metabolism, nicotinate and nicotinamide metabolism, arginine and proline metabolism, glycine, serine and threonine metabolism, aminoacyl-tRNA biosynthesis, nitrogen metabolism, phenylalanine metabolism, methane metabolism, phenylalanine, tyrosine and tryptophan biosynthesis, and D-Glutamine and D-glutamate metabolism (Figure 8A). In negative ion mode, the main metabolic pathways significantly affected by CKD included citrate cycle (TCA cycle), histidine metabolism, glyoxylate and dicarboxylate metabolism, glycine, serine and threonine metabolism, phenylalanine, tyrosine and tryptophan biosynthesis, alanine, aspartate and glutamate metabolism, biosynthesis of unsaturated fatty acids, D-Glutamine and D-glutamate metabolism, and linoleic acid metabolism (Figure 8B). In positive ion mode, the main metabolic pathways significantly affected by AM and CZ treatment included glycine, serine and threonine metabolism, nicotinate and nicotinamide metabolism, biotin metabolism, vitamin B6 metabolism, methane metabolism, fatty acid elongation in mitochondria, and fatty acid metabolism (Figure 8C). In negative ion mode, the main metabolic pathways significantly affected by AM and CZ treatment included alanine, aspartate and glutamate metabolism, glutathione metabolism, cyanoamino acid metabolism, arginine and proline metabolism, glycine, serine and threonine metabolism, aminoacyl-tRNA biosynthesis, citrate cycle (TCA cycle), nitrogen metabolism, methane metabolism, phenylalanine, tyrosine and tryptophan biosynthesis, D-Glutamine and D-glutamate metabolism, linoleic acid metabolism, ascorbate and aldarate metabolism (Figure 8D).

**FIGURE 9:**
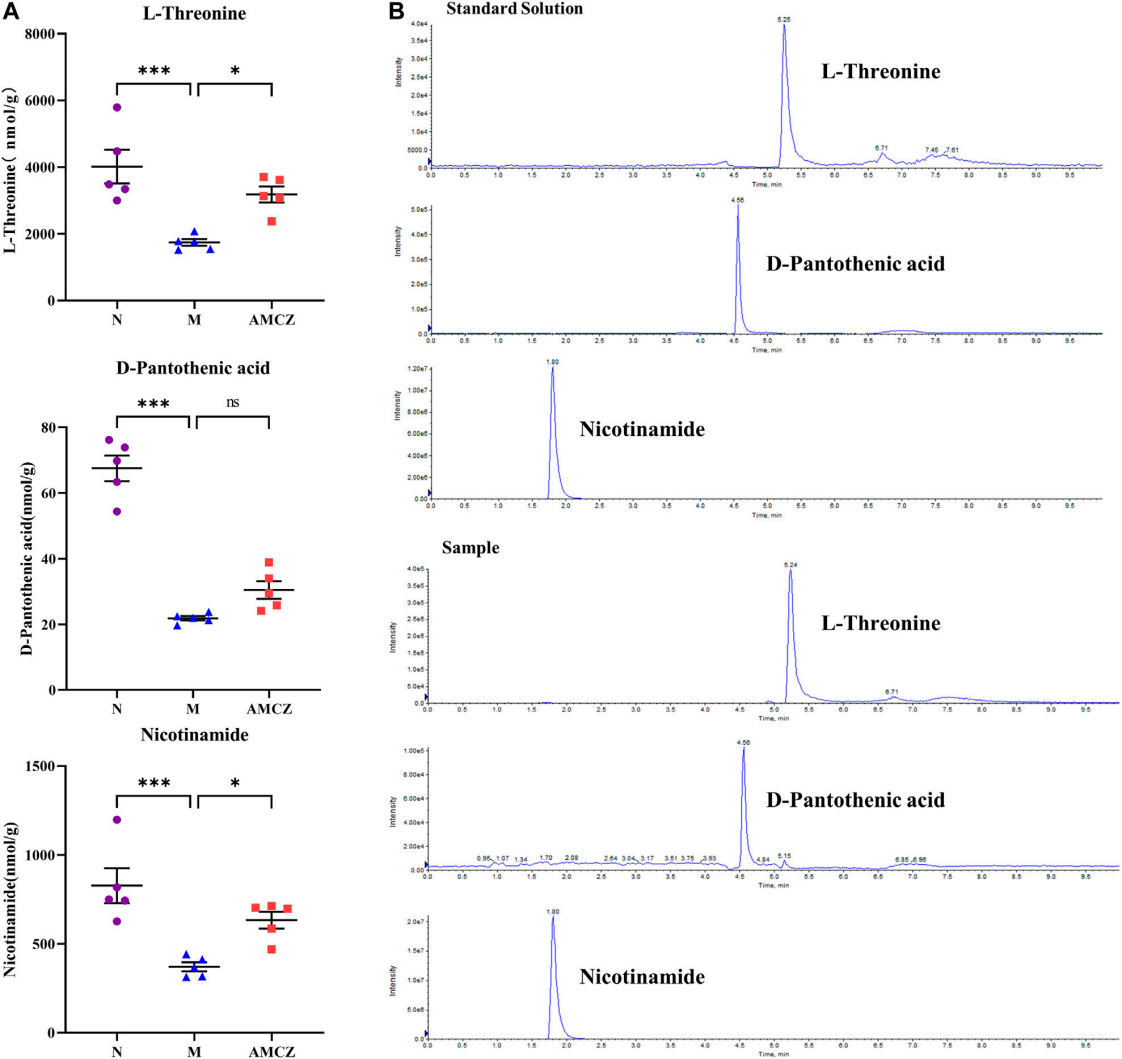
AM and CZ upregulation the concentration of L-Threonine, D-pantothenic acid, and nicotinamide. **(A)** The concentration of L-Threonine, D-pantothenic acid, and nicotinamide in three groups (n = 5). Data are presented as the means ± SEM (***p < 0.001, *p < 0.05). **(B)** The extracted ion chromatographs (EICs) from a standard solution, and a sample of the targeted analytes under optimal conditions.

We obtained massive data through metabolomics. In order to obtain marker metabolites, we analyzed metabolomics in two different ways: up-down (upregulated in M and downregulated by treatment) and down-up (downregulated in M and upregulated by treatment). Furthermore, we screened L-Threonine, D-pantothenic acid, and nicotinamide as potential marker metabolites by using a random forest model. In order to observe the effects of AM and CZ on potential marker metabolites, UHPLC-MRM-MS/MS analysis was performed to determine the concentration of L-threonine, D-pantothenic acid, and nicotinamide (Figure 9). It was found that the concentration of L-Threonine, D-pantothenic acid, and nicotinamide were decreased in M group compared with N group (*p* < 0.001), and the concentration of L-Threonine, D-pantothenic acid had a significant increased was observed in AMCZ group compared with M group (*p* < 0.05) (Figure 9A). The concentration of D-pantothenic was increased in AMCZ group compared with M group, but there was no statistical difference.

#### Effects of AMCZ on SIRT1/JNK signaling pathway in CKD rats

**FIGURE 10:**
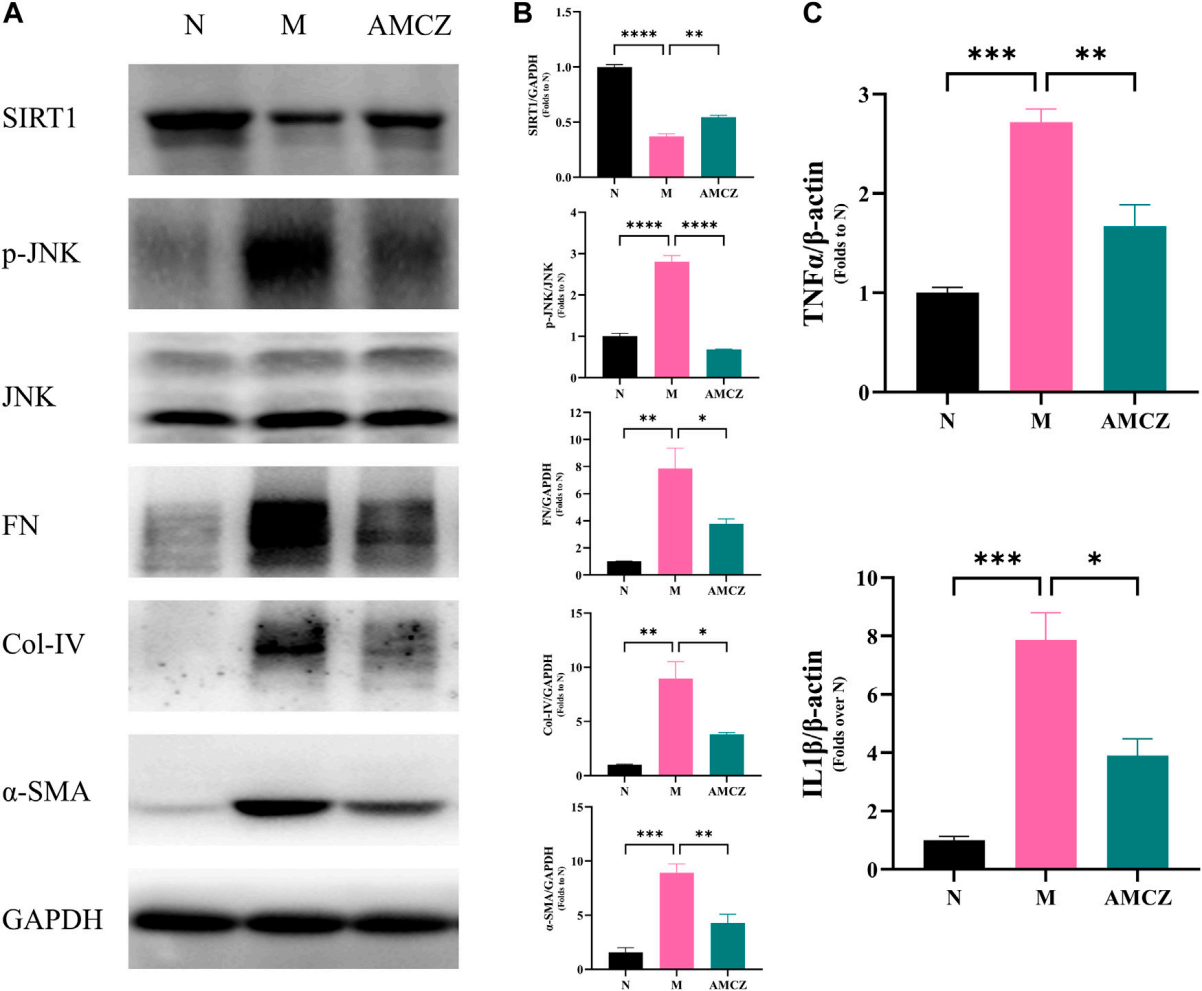
Effects of AM and CZ on SIRT1/JNK signaling pathway in adenine-induced CKD rats. **(A)** Representative Western blot images of SIRT1, p-JNK, JNK, FN, Col-IV, and α-SMA expression in the kidneys of rats. **(B)** Densitometric analysis of SIRT1, p-JNK, JNK, FN, Col-IV, and α-SMA normalized to GAPDH content. Values are expressed as mean ± SEM,n = 3 rats per group (*p < 0.05 **p < 0.01,***p < 0.001, and ****p < 0.0001 between the indicated two groups). The total RNA was isolated and subjected to real time RT-PCR to analyze the products of pro-inflammatory cytokines, IL-1β and TNF-α in cortex tissues treated with AM and CZ. β-actin was used as an internal control (*p < 0.05 **p < 0.01, and ***p < 0.001 between the indicated two groups).

Nicotinamide adenine dinucleotide (NAD^+^), is a key signaling molecule that regulates intermediary metabolism. NAD^+^ acts as a coenzyme in the redox reaction and serves as an important substrate of sirtuins (Sirts) (Verdin, 2015, 1). We further examined the activation of SIRT1/JNK signaling pathway in the kidneys of CKD rats with AMCZ. As shown in Figure 10A, B, compared with the N group, the expression of SIRT1 was significantly downregulated in M group (*p* < 0.01). AMCZ attenuated this trend (*p* < 0.01). Compared with the N group, the expression of p-JNK was significantly upregulated in M group (*p* < 0.01). This increase was reversed by AM and CZ treatment of the CKD rats (*p* < 0.01).

#### Effects of AMCZ in the regulation of inflammation in CKD rats

The dysregulated inflammation is an important mechanism in the development of CKD. SIRT1 and JNK phosphorylation have been demonstrated to be crucial in modulating inflammation (Papaconstantinou, 2019; Yang et al., 2022). To further detect the level of pro-inflammatory factors, qRT-PCR was performed to assess the expression of IL-1β and TNF-α in cortex tissues. The results (Figure 10C) showed that expression of IL-1β and TNF-α in the M group was higher than that in the N group (*p* < 0.05), while AMCZ administration reversed the increase (*p* < 0.05).

#### Effects of AMCZ on renal fibrosis in CKD rats

The increased expressions of FN, Col-IV and α-SMA are crucial signals of renal tubulointerstitial fibrosis in the CKD progression. Figure 10 displayed the anti-fibrotic effects of the AMCZ treatments. Compared with the M group, the expression of α-SMA was significantly downregulated by AMCZ (*p* < 0.01). As shown in Figure 10A, B, 3 weeks of adenine-administration resulted in a significantly increased expression of Col-IV and FN (*p* < 0.01). AMCZ attenuated this trend (*p* < 0.05).

## Discussion

In this study, we found histidine metabolism, nicotinate and nicotinamide metabolism, arginine and proline metabolism, glycine, serine and threonine metabolism were correlated with CKD pathophysiology. Additionally, we studied the effect and possible mechanisms of AM and CZ in the treatment of CKD, the results showed that AM and CZ alleviated adenine-induced kidney dysfunction, inflammation, and renal fibrosis, which are related to SIRT1/JNK signaling pathway. Furthermore, L-Threonine, D-pantothenic acid, and nicotinamide may be potential biomarkers for the progression and treatment of CKD.

As a progressive disease, CKD is characterized by dysfunctions in renal structure and function, which can be caused by various factors. Currently, the exact mechanism of CKD is still unclear (Lin et al., 2019). CKD progression may be attributed to parenchymal cell loss, microvascular damage, metabolic changes, oxidative stress, chronic inflammation, fibrosis, and reduced renal regeneration (Ruiz-Ortega et al., 2020). Studies have demonstrated that patients with CKD have significantly lower plasma histidine concentrations and negative correlations with pro-inflammatory markers (Watanabe et al., 2008), whereas histidine supplementation results in several health benefits, including anti-inflammatory, antioxidant, glucose-regulatory, and weight management (Holeček, 2020; Thalacker-Mercer and Gheller, 2020). Similarly, in our research, we found that L-histidine and L-glutamic acid were lower in the M group, and the expression of IL-1β and TNF-α in the M group was higher then N group. Histidine metabolism may be one of the main metabolic pathways significantly affected by CKD. Moreover, we found that nicotinamide metabolism (including nicotinamide adenine dinucleotide (NAD), nicotinamide adenine dinucleotide phosphate (NADP), niacinamide, and nicotinamide ribotide) was lower in the model group, which was similar to Zhu et al. (Zhu et al., 2021). Arginine is a semi-essential amino acid that is synthesized from glutamine, glutamate, and proline, and it is degraded by many pathways (Popolo et al., 2014). One of these breakdown mechanisms is the conversion of arginine to urea and ornithine. Additionally, arginine can be metabolized to nitric oxide (NO) and citrulline in the cytosol by nitric oxide synthetase (NOS). It has been reported that CKD patients produce less NO (Wever et al., 1999). In this study, we found D-proline and L-proline were lower in the CKD group.

There was a report that streptozotocin-induced diabetic mice have increased levels of urinary glycine (Hyeon et al., 2020), and elevated glycine levels in the urine are as a marker of tubular damage in rats (Wen et al., 2011). Glycine is the simplest amino acid, and its tiny size allows it to pass through the glomerular filtrate barrier. There is considerable resorption of this substance by the proximal tubules, and it is only detected in trace amounts in normal urine. Feng et al. (Feng et al., 2019) found serum glycine, cinnamoylglycine, and phenylacetylglycine were increased in CKD rats and renal function decline was associated with glycine metabolism. However, we found renal glycine levels were lower in CKD rats, but cinnamoylglycine and phenylacetylglycine levels were higher. This could be different from the test samples, therefore more research is needed.

We observed that levels of Scr and BUN, which were elevated in CKD, decreased after AM and CZ administration. Glycine, serine, and threonine metabolism and nicotinate and nicotinamide metabolism exhibited the greatest response to AM and CZ. Interestingly, Nicotinate and nicotinamide metabolism, and Glycine, serine and threonine metabolism were identified in both N versus M and M versus AMCZ groups. Moreover, we found that the concentration of nicotinamide was decreased in M group compared with N group, and an increased was observed in AMCZ group measured by UHPLC-MRM-MS/MS analysis. Nicotinamide, a vitamin B3 amide and precursor of nicotinamide adenine dinucleotide (NAD^+^), is a powerful antioxidant that may substantially minimize the damage produced by reactive oxygen species (ROS) in cells under oxidative stress (Mejía et al., 2018). Using UHPLC-MS/MS analysis, we found that in the M group, levels of 1-(beta-D-ribofuranosyl)-1, 4-dihydronicotinamide were significantly decreased, but they increased following administration of AM and CZ. 1-(beta-D-ribofuranosyl)-1, 4-dihydronicotinamide, a reduced form of niacinamide riboside, is considered to be a precursor to NAD^+^(Bogan and Brenner, 2008). NAD^+^ serves as a substrate of SIRT1 and regulates SIRT1 expression (Mouchiroud et al., 2013). It has been demonstrated that NAD^+^ levels and SIRT1 activity can be effectively increased by administering NAD^+^ precursors such as nicotinamide mononucleotide (Yoshino et al., 2011) and nicotinamide riboside (Cantó et al., 2012). It has been reported that nicotinamide is involved in the regulation of inflammation (Weiss et al., 2015; Zheng et al., 2019). SIRT1 and JNK phosphorylation have been demonstrated to be crucial in modulating inflammation. In the present study, we found that the low expression of 1-(beta-D-ribofuranosyl)-1,4-dihydronicotinamide, nicotinamide, and SIRT1 was improved by the AMCZ treatment. AMCZ markedly lowered the elevated levels of p-JNK, FN, Col-IV, α-SMA, IL-1β, and TNF-α in CKD rats, suggesting AMCZ attenuated renal fibrosis and inflammation through the modulation of SIRT1/JNK signaling pathway.

Besides, D-pantothenic acid is another potential marker worthy of attention. It has been proposed that pantothenic acid could be a potential biomarker for CKD progression. Furthermore, pantothenic acid is being used as a biomarker of albuminuria in normoalbuminuric hypertensive patients and has also been shown to be a promising biomarker for predicting diabetes-induced kidney impairment (Gonzalez-Calero et al., 2016; Ma et al., 2020). In addition, pantothenic acid is a potential therapeutic target for DN treatment (Daugherty et al., 2002; Demirci et al., 2014). Here, we showed that the concentration of D-pantothenic acid were decreased in M group compared with N group, and an increased were observed in AMCZ group, suggesting that D-pantothenic acid were one of renal protective mechanisms.

Notably, L-threonine, an essential amino acid in both humans and animals, was decreased in CKD and returned to control levels in response to AM and CZ. Pathway enrichment analysis showed that Glycine, serine and threonine metabolism might be related to the occurrence and development of CKD. Recent research found that L-threonine can reduce inflammatory cytokine expression (Wang et al., 2021). L-threonine could potentially play a role in the detection and treatment of CKD. However, few studies have examined the relationship between L-threonine and CKD.

## Conclusion

In summary, our study revealed that after AM and CZ treatments, CKD rats experienced a slew of complex and serious metabolic disorders. In addition, we discovered significant differences in metabolism in CKD rats, including nicotinate and nicotinamide metabolism, arginine and proline metabolism, and glycine, serine, and threonine metabolism. Moreover, we found that AM and CZ significantly reduced renal fibrosis and inflammation in CKD rats, which may be related to the regulation of SIRT1/JNK signaling pathway. Furthermore, L-Threonine, D-pantothenic acid, and nicotinamide may be potential biomarkers for the progression and treatment of CKD. In conclusion, the results described above may contribute to the identification of metabolic biomarkers and novel therapeutic strategies to prevent or delay the progression of CKD.

## Data Availability

The original contributions presented in the study are included in the article/Supplementary Material, further inquiries can be directed to the corresponding authors.

## Supplementary material

Supplementary Figure S1 The extracted ion chromatogram (EIC) of QC samples obtained from in positive **(A)** and negative **(B)** ion mode.

Supplementary Figure S2 Permutation test of OPLS-DA model for N versus M in positive **(A)** and negative **(B)** ion mode. Permutation test of OPLS-DA model for M versus AMCZ in positive **(C)** and negative **(D)** ion mode.

Supplementary Table S1 Significantly differentially expressed metabolites in the kidney of N versus M in positive ion mode.

Supplementary Table S2 Significantly differentially expressed metabolites in the kidney of N versus M in negative ion mode.

Supplementary Table S3 Significantly differentially expressed metabolites in M and AMCZ in positive ion mode.

Supplementary Table S4 Significantly differentially expressed metabolites in M and AMCZ in negative ion mode.

## Abbreviations

ACEI, angiotensin converting enzyme inhibitor; AM, *Astragalus membranaceus*; ARB, angiotensin receptor blocker; BUN, blood urea nitrogen; CKD, chronic kidney disease; CZ, *Curcuma zedoaria*; ESI, electrospray ionization; NAD+, nicotinamide adenine dinucleotide; NADP, nicotinamide adenine dinucleotide phosphate; NO, nitric oxide; NOS, nitric oxide synthetase; OPLS-DA, orthogonal partial least squares discriminant analysis; PCA, principal component analysis; QC, quality control; SCR, serum creatinine; SGLT2, sodium-glucose cotransporter 2; TCM, traditional chinese medicine; UHPLC-MS/MS, ultra-high-performance liquid chromatography-tandem mass spectrometry; VIP, variable importance in the projection.
