# Supplementary figures and images for: Biomarker identification and pathway analysis of Astragalus membranaceus and Curcuma zedoaria couplet medicines on adenine-induced chronic kidney disease in rats based on metabolomics
Source: Front Pharmacol. 2023 Apr 6;14:1103527. doi: 10.3389/fphar.2023.1103527 (PMC10116179; doi:10.3389/fphar.2023.1103527)

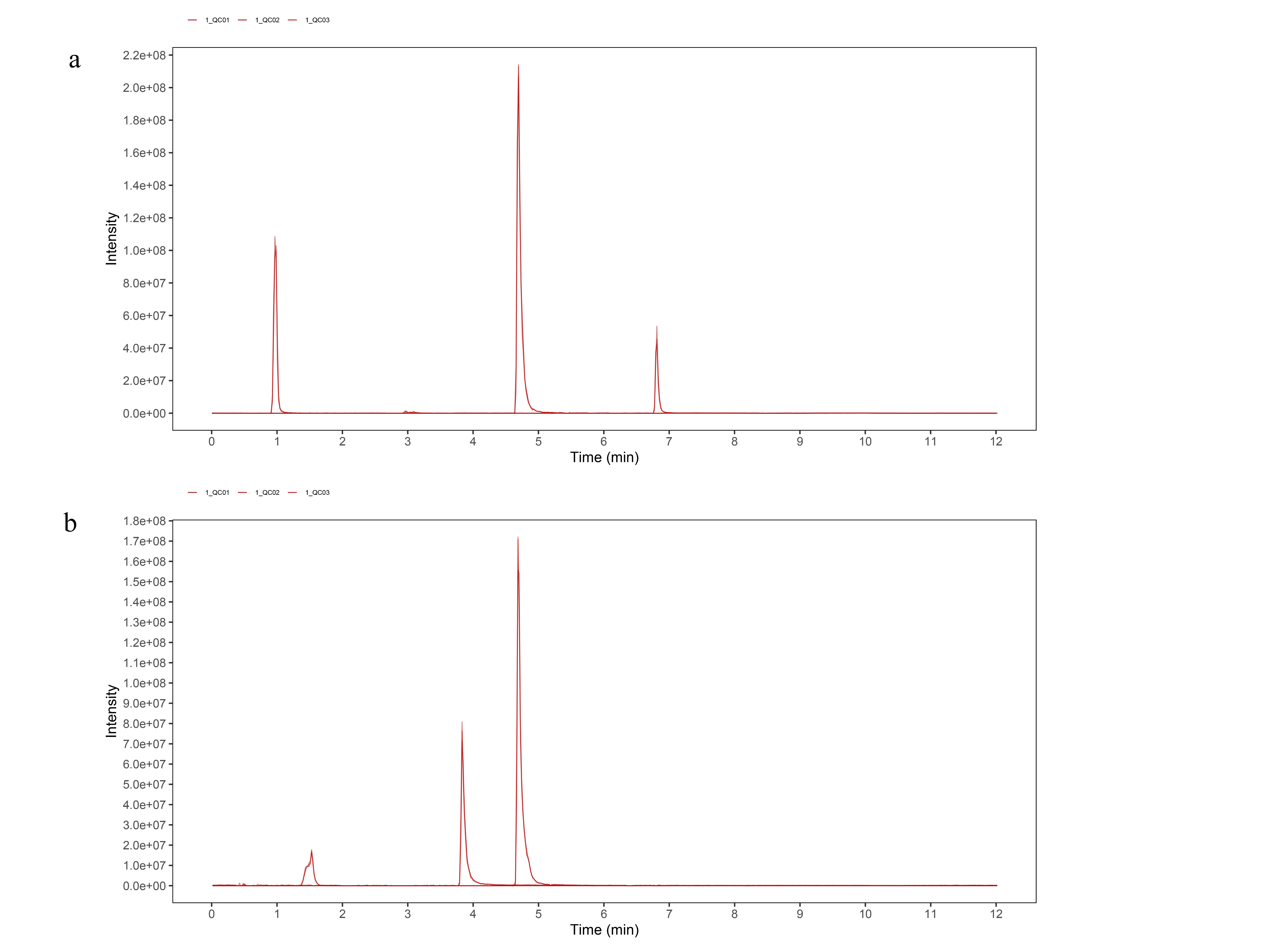

Supplement: Supplementary file 4 [file Image1.TIF]
